# Assessment of Dietary Patterns Represents a Potential, Yet Variable, Measure of Inflammatory Status: A Review and Update

**DOI:** 10.1155/2019/3102870

**Published:** 2019-01-20

**Authors:** Mariana C. Calle, Catherine J. Andersen

**Affiliations:** ^1^Health Sciences Department, Worcester State University, Worcester, MA 01602, USA; ^2^Department of Biology, Fairfield University, Fairfield, CT 06824, USA

## Abstract

Chronic low-grade, systemic inflammation is a well-characterized risk factor in the development of chronic metabolic diseases, such as cardiovascular disease, type 2 diabetes, and metabolic syndrome. Diet could be an effective strategy for reducing inflammation associated with chronic disease. While anti-inflammatory properties of isolated dietary bioactive and functional foods have been routinely studied, the evaluation of dietary patterns on inflammation warrants further review—especially given the recent inclusion of dietary pattern recommendations into dietary guidelines and policies. Therefore, the objective of this narrative review is to examine current evidence linking diet to low-grade, systemic inflammation within the context of chronic disease. Specifically, we provide an update on the findings from human trials that have characterized anti-inflammatory properties of dietary patterns, defined by various methods and indexes. Given the complexity of interpreting results from dietary pattern analysis, we further present recent evidence on the anti-inflammatory roles of isolated bioactive nutrients and functional foods that are common components of distinct dietary patterns, in addition to considerations for interpreting dietary pattern research, population-specific dietary recommendations, and future studies. Overall, we observe a vast range of variability in the evidence from observational studies that have evaluated the relationships between healthy dietary patterns and inflammatory markers. These studies highlight the need for additional intervention studies with study designs that account for metabolic status, diversity in populations, breadth of inflammatory measurements, fasting vs. postprandial effects of diet, and control of confounding factors (e.g., genotype, microbiome profiles, and dietary adherence) in order to better understand the effect that diet has, as a whole, on inflammation. These strategies will help to strengthen diet recommendations aimed at reducing inflammation and chronic disease risk.

## 1. Introduction

Chronic low-grade, systemic inflammation is a distinctive feature present in the development of metabolic and cardiovascular diseases (CVD) [1–4]. Considering the mounting health care cost [5] and prevalence [6] of chronic diseases, it is essential to better understand how to ameliorate inflammation.

Low-grade, systemic inflammation is characterized by a two to threefold increase in concentrations of cytokines in the bloodstream, such as tumor necrosis factor alpha (TNF-*α*), interleukin-6 (IL-6) [7], and high-sensitivity C-reactive protein (hsCRP)—a well-established biomarker of inflammation and CVD risk [8]. Additionally, other interleukins (e.g., IL-1*β*, IL-4, IL-8, and IL-10), and adhesion molecules (e.g., vascular cell adhesion molecule 1 (VCAM1) and intracellular adhesion molecule 1 (ICAM1)) also contribute to the determination of an individual's inflammatory status [9, 10].

The origins of low-grade, systemic inflammation are multifactorial and often stem from obesity-induced metabolic tissue dysfunction and/or the failure of immune cells to adequately ameliorate proinflammatory responses [11]. The production of inflammatory mediators is an essential mechanism by which leukocytes confer immune protection in response to infectious pathogens and tissue injury. Acute inflammatory responses begin with the activation of innate immune cells (e.g., macrophages, monocytes, and granulocytes), followed by highly specific and coordinated activation of adaptive immune cell populations (e.g., T and B lymphocytes) [12, 13]. Upon clearance of the initial immune insult, subsequent production of anti-inflammatory cytokines and eicosanoids is critical to ensure resolution of inflammation, prevent prolonged proinflammatory responses, promote tissue healing, and facilitate a return to homeostatic tissue function [14]. In obesity, excessive weight gain leads to adipose tissue remodeling, which includes increases in adipocyte hypertrophy, hypoxia, stress, and apoptosis/necrosis [15]. Activation and recruitment of proinflammatory leukocytes perpetuate the prolonged production of inflammatory mediators, whereas the subsequent release of adipose-derived proinflammatory cytokines and free fatty acids into the circulation can lead to widespread metabolic dysfunction [16]. Together, systemic disturbances in metabolism and tissue health promote chronic low-grade inflammation and increased risk for chronic disease [17].

Dietary intake modulates inflammation and represents an effective and promising therapeutic target to reduce metabolic dysfunction and chronic disease risk [17–19]. The effect of diet on inflammation has been extensively analyzed by studying isolated bioactive nutrients (fiber [20], fatty acids [21], and polyphenols [22, 23]) and functional whole foods (eggs [24] and blueberries [25]). However, recent studies highlight the value of characterizing the relationship between diet and inflammation through assessment of dietary patterns [11, 26, 27]. A dietary pattern can be described as a comprehensive dietary evaluation in which multiple foods and/or nutrients are examined collectively [28]. Studying dietary patterns can be complex and challenging, in part due to the diversity of patterns within different cultures and populations, in addition to the inherent heterogeneity of physiological responses to diet due to variability in genetics, microbiome profiles, metabolic status, etc. [29]. However, dietary patterns additionally have a great potential for translation to the general public, as evidenced by the inclusion of dietary pattern recommendations in the *2015-2020 Dietary Guidelines for Americans* [23].

The objective of the present narrative review is to examine current evidence linking diet to low-grade, systemic inflammation within the context of chronic disease. Specifically, we provide an update on the findings from human trials within the past 5 years (2014–present) that have characterized anti-inflammatory properties of dietary patterns, defined by various methods and indexes. Given the complexity of interpreting results from dietary pattern analysis, we further present recent evidence on the anti-inflammatory roles of isolated bioactive nutrients and functional foods that are common components of distinct dietary patterns, in addition to considerations for interpreting dietary pattern research, population-specific dietary recommendations, and future studies.

The literature search performed in Scopus and PubMed included the following keywords and its combinations: dietary patterns, food patterns, diet, dietary inflammatory index, foods, anti-inflammatory foods, anti-inflammatory nutrients/inflammation, inflammatory markers, CRP, biomarkers of inflammation, cytokines, low-grade, systemic inflammation, and adipokines. The criteria encompassed the inclusion of only peer-reviewed human studies initially from 2016 to present time. Then, a second search expanded the years from 2014 to present to provide a more comprehensive assessment of dietary patterns within the context of inflammatory biomarkers and chronic disease.

## 2. Evaluation of Inflammatory Dietary Patterns Utilizing Dietary Indexes

In order to evaluate diet quality in relation to inflammation, researchers have developed various methods to establish measurable dietary patterns. Reedy et al. [29] refer to dietary patterns as a way of conceptualizing numerous diet exposures as a multidimensional construct. For example, Western-style dietary patterns that are rich in refined carbohydrates, sodium, and trans- and saturated fatty acids (SFA) have additionally been linked with higher levels of inflammatory markers, which in turn increase CVD risk [30–32]. Conversely, Mediterranean-style dietary patterns that are rich in fiber, lean protein, monounsaturated (MUFA), and omega-3 polyunsaturated fatty acids (PUFA) are anti-inflammatory and cardioprotective [33–35].

Dietary patterns can be derived using principal component analysis, reduced rank regression, and index-based methods, such as the Healthy Eating Index (HEI) score, the Empirical Dietary Inflammatory Index (EDII) [36], or the Diet Inflammatory Index (DII) [37]. The HEI compares a given diet to the American dietary guidelines as the gold standard [38]. The EDII and, the more widely used, DII are *a posteriori* approaches to examine the inflammatory potential of a diet [36]. Each method provides a unique prospective in evaluating the effects of diet on inflammation.

### 2.1. Healthy Eating Index (HEI) Score

A better quality diet, as shown with adherence to dietary guidelines, is associated with lower inflammatory markers, data from the Women's Health Initiative study [39] and data from the Malmo Diet and Cancer study [40]. Similarly, an observational cohort in South America used the principal component analysis to compare 2 dietary patterns. The “prudent” dietary pattern, characterized by a higher intake of fruits, vegetables, seafood, whole grain cereals, and low-fat dairy products, was associated with reduced plasma CRP for men compared to the “Western” (standard U.S.) dietary pattern [32]. Researchers calculated the HEI for an 8-week weight loss intervention in obese people diagnosed with metabolic syndrome [41]. Changes in the HEI were associated with changes in gene expression of inflammatory markers [41, 42].

### 2.2. Empirical Dietary Inflammatory Index (EDII)

The EDII uses reduced rank regression to derive a dietary pattern based on food groups. These food groups were positively associated with inflammatory markers, a.k.a. “proinflammatory”: processed meat, red meat, other vegetable-refined grains, high-energy beverages, low-energy beverages, and tomatoes and the following were inversely associated: dark yellow vegetables, leafy green vegetables, snacks, fruit juice, pizza, beer, wine, tea, and coffee. In the study of dietary patterns, it is the combination of these food groups, not each of them individually, that reveal these associations [36]. This index was validated using the data from the Nurses' Health Study I (NHS), NHS-II, and the Health Professionals Follow-Up Study, with only male subjects [36]. Tabung et al. [36] reported stronger associations between the EDII and IL-6, CRP, and the overall inflammatory marker scores in men of normal weight when compared to overweight/obese men. The authors suggested that the EDII, which they also called the Empirical Diet Inflammatory Pattern (EDIP), needs to be reproduced in multiethnic/multiracial populations [36]. Thus, the same research group [43] performed a cross-sectional study using the data from the Women's Health Initiative observational study and calculated EDIP scores using baseline food frequency questionnaire data from 31,472 women, aged 50-79 yrs. Authors reported that independent of energy intake, BMI, and physical activity, higher EDIP scores were significantly associated with higher (lower for adiponectin) absolute concentrations of all inflammatory biomarkers (CRP, IL-6, TNF-*α*, and TNF-*α* receptors 1 and 2) [43]. In a report by Soltani et al. [44], a cross-sectional study performed in 403 middle-agedIranians used the same method to examine the relationship between an “inflammatory dietary pattern” and the odds of an unhealthy phenotype among overweight/obese adults. Authors concluded that there were associations between the “inflammatory dietary pattern” and the unhealthy phenotype (high fasting blood sugar, and low-HDL-C), but that they were dependent on the energy intake [44].

### 2.3. Diet Inflammatory Index (DII)

The Dietary Inflammatory Index (DII) uses a principal component analysis to categorize an individual's diet as anti- or proinflammatory, based on the capacity of diets to modulate systemic inflammatory biomarkers [37]. Food parameters were assigned scores based on research (1943 articles) describing the relationship between those parameters and inflammation. DII was validated with the data from the Asklepios Study [42], NHANES 2005-10 [45], and the Northern Sweden Health and Disease Study [46]. A proinflammatory diet (based on the DII) is associated with increased all-cause mortality according to a recent report from two European large cohort studies, the SUN and PREDIMED trials [47]. Recently (2018), the association of DII with depressive symptoms remained after the Framingham Risk Score (FRS) adjustment, suggesting a relationship between the inflammatory potential of the diet and depression [48]. Phillips et al. [49] reported that individuals with higher Energy-adjusted DII (E-DII) scores displayed several features of the metabolic syndrome (MetS). Similarly, the higher inflammatory potential of diet denoted higher values of serum lipids, CRP and kidney function tests, and a higher EuroSCORE as a predictor of postoperative mortality among patients who are candidates for coronary artery bypass grafting (CABG) [50]. Taken together, there are several contemporary reports of studies using the DII scores to improve the understanding of dietary patterns and inflammation in healthy and diseased individuals.

### 2.4. Mediterranean-Style Dietary Patterns

Concerning the type of diet, there is mounting epidemiological evidence that specific dietary patterns, such as adherence to Mediterranean-style dietary patterns, are anti-inflammatory and concomitantly protective against CVD. Additionally, this diet can also benefit coronary artery disease (CAD) patients [35, 51, 52].

Waldeyer et al. [35] analyzed adherence to the Mediterranean diet using the INTERCATH cohort data. This cohort consisted of 70% of participants being male, diagnosed with CAD, and aged 61-77 yrs. A higher adherence was independently associated with less CAD severity. Supporting the anti-inflammatory role of the diet, hsCRP inversely correlated with adherence to the Mediterranean diet. Conversely, a 6-month intervention trial testing the Mediterranean diet vs. a low-fat diet did not result in decreases in CRP or IL-6 for Australians diagnosed with CAD [51]. Researchers calculated the DII score for each diet, and those following the Mediterranean diet improved by lowering their DII score (the lower, the more anti-inflammatory). The small sample size, (*n* = 27 per group) and the fact that some participants had a normal hsCRP at baseline, could contribute to the discrepancy in translating population-based effects into clinical changes in plasma biomarkers.

The Moli-sani study (cohort of 24,325 men and women) showed that adherence to a “Mediterranean-like Diet” was associated with lower hsCRP, blood platelets, and white blood cell counts [53]. On the other hand, adherence to a DASH diet, but not the Mediterranean diet, was associated with lower hsCRP in a group of 320 overweight Iranian females [52]. There are some similarities within these diets, specifically related to the emphasis in fruits and vegetables.

## 3. Isolated Bioactive Nutrients and Functional Foods

As described above, the impact of dietary patterns in modulating inflammation is due to complex interactions between functional foods and nutrients with bioactive properties. Accordingly, it is important to evaluate the effects of isolated nutrients, macronutrient distributions, and whole foods that are common components of distinct dietary patterns in order to characterize the contributions of each dietary factor. This information may be important in predicting the effects of dietary patterns in cases where dietary allergies, intolerances, or food preferences may prevent individuals from consuming the full range of components that comprises a distinct dietary pattern. In this section, we present recent studies that evaluate the effect of one macronutrient, which in turn affects macronutrient distribution (Table 1). We further review recent studies that analyze the effects of isolated nutrients and whole food components, such as fiber, phospholipids, SFA, omega-3 PUFA, and polyphenol-rich extracts (Table 2).

### 3.1. Dietary Macronutrient Distribution and Inflammation

Modulation of macronutrient ratios is a wide-reaching and impactful dietary variable to test. A cross-sectional study performed on 1785 people with type 2 diabetes mellitus (T2DM) explored the association of metabolic risk factors vs. fat and carbohydrate proportions of the diet. The authors reported that a fiber intake ≥ 15 g/1000 kcal was associated with lower CRP, whereas added sugar consumption higher than 10% was associated with higher CRP [54]. Increased fat intake was additionally associated with higher CRP; however, authors did not include a description of the type of the dietary fat (MUFA vs. PUFA vs. SFA) [54]. This study had limitations in that the researchers set arbitrary macronutrient cutoffs for tertile analysis, leading to drastically different sample sizes between macronutrient intake groups. The authors divided the macronutrients of interest into arbitrary categories, the cutoff points were <25%, 25–34%, or ≥35% for total fat intake; <45%, 45–59%, or ≥60% for CHO; <10, 10–14, or ≥15 g/1000 kcal for fiber; and <5%, 5–9%, or ≥10% for added sugar. In the extreme of the tertiles, e.g., group of >60% CHO (*n* = 12) and for fat <25% (*n* = 15) vs. the other groups that had hundreds of participants. Additional studies have observed significant reductions in inflammatory markers in T2DM and coronary heart disease populations following carbohydrate-restricted diets (which are consequently rich in fat and protein), although these findings may be confounded by concurrent weight loss [55, 56].

### 3.2. Effects of Bioactive Nutrients and Functional Foods on Inflammation

Beyond macronutrient distribution, bioactive compounds provided in isolated forms, or as components of functional foods, further contribute to the pro and anti-inflammatory properties of dietary patterns. A 6-week intervention of omega-3 PUFA supplementation provided as 5 g/day of fish oil did not have an impact on inflammatory biomarker levels in overweight participants [62]. Meanwhile, a randomized, controlled trial (RCT) evaluating the effect of 30-day polyphenol-rich pomegranate extract supplementation in overweight and obese individuals resulted in weight loss and reductions in IL-6, CRP, and malondialdehyde (MDA) [22]. Thus, body fat reduction may be a confounding factor in determining the effects of the supplement. In healthy people, a crossover trial tested the postprandial effects of one Mediterranean meal that was rich in tocopherol vs. a “Western meal” high in fat on mRNA expression of genes involved in the inflammasome pathway [63]. Researchers reported an upregulation of inflammasome gene expression following consumption of the high-fat meal. Similarly, changes in fasting plasma biomarkers were reported in another study after 4 weeks of consuming an isocaloric Mediterranean vs. high-fat breakfast in a group of overweight individuals [64]. Results from these studies illustrate the importance of including postprandial testing when evaluating the effect of foods. Adding antioxidants, such as polyphenols, to a high-fat meal could attenuate the postprandial inflammatory response; however, a crossover study testing polyphenol supplementation in healthy individuals failed to show this protective effect [25].

## 4. Considerations for Population-Specific Dietary Recommendations and Future Studies

Most results from epidemiological and cross-sectional studies showed associations between diet quality and inflammatory markers. Conversely, findings from the recent RCTs highlighted in this review show discrepancies in changes of fasting plasma cytokines in healthy [59], obese [41, 57], diabetic [55, 56], and CHD [56] individuals in response to diet. In the following section, we provide a critical analysis of factors that may contribute to the variability across studies and highlight considerations for study design when evaluating dietary patterns moving forward.

### 4.1. Metabolic Status

Obese individuals and/or persons with metabolic abnormalities, including diabetes or metabolic syndrome (MetS), appear to be the main target population to study the effect of diet and inflammation. The response to a meal challenge, such as a “traditional” Brazilian SFA-rich breakfast vs. a breakfast of highly unsaturated fats and fiber, differs based on the metabolic status of people with MetS [64]. The authors concluded the need for using different biomarkers when examining responses to dietary interventions of individuals at different levels of CVD risk. As an example, an 8-week weight loss intervention conducted with obese MetS participants resulted in changes in plasma MDA and plasminogen activator inhibitor-1 (PAI-1), but additionally did not show decreases in CRP and IL-6 [41]. Conversely, weight loss in overweight individuals without a MetS classification has been shown to lower CRP [65]. To further illustrate the complexity of how the same food could affect individuals differently, in a comprehensive review about bioactive egg components and inflammation, we have previously reported that the majority of research suggests that egg intake promotes a neutral or proinflammatory response in healthy adults, whereas those with metabolic abnormalities have either an anti-inflammatory or a neutral effect. It is possible that this variation is attributable to differences in intestinal absorption of dietary cholesterol, or other factors such as the composition of the microbiome or genetic variation [24].

As mentioned above, people with metabolic disorders participated in the majority of the studies evaluating associations of diet with inflammatory biomarkers [41, 51, 54, 61]. It appears that if low-grade, systemic inflammation is present, then certain modifications in the diet could ameliorate it; however, this is not as clear with regard to prevention of inflammation. There were few studies done in healthy individuals and the responses, as expected, were different from those seen in people with metabolic disorders. The majority of studies analyzed in the present review conducted with healthy individuals show no effects of dietary patterns and individual components on inflammatory markers. One possible reason could be that the cytokines were not elevated to begin with and/or that the dietary challenges did not cause a significant inflammatory response in these populations due to differences in genetics, microbiome profiles, and overall metabolic and immune health. In healthy people with normal levels of inflammatory markers, a short diet intervention (6 weeks) did not change plasma inflammatory markers [59]. Similarly, there were no changes in plasma IL-6, CRP, or TNF-*α* in a group of 191 overweight individuals receiving omega-3 PUFA supplementation for 6 weeks [62]. Meanwhile, some studies performed in healthy people in other individual nutrients reported improvement such as blueberries [25], pomegranates [22], and tocopherol-enriched meals [63].

### 4.2. Need for Comprehensive Inflammatory Biomarker Assessment

Discrepancies in diet-induced effects on inflammation may further be attributed to differences in measured biomarkers. A review by Calder et al. [66] discussed the importance of measuring many biomarkers to capture changes, since there may be specific inflammatory markers associated with each chronic disease. For example, Van Bussel et al. [67] used a combination of biomarkers to generate a “low-grade inflammatory score” in order to examine associations between food groups and inflammation in a subset of 557 participants with increased risk of CAD within the CODAM (Cohort of Diabetes and Atherosclerosis Maastricht) observational study. Consumption of vegetables, fruit, wine (in moderation), and poultry, as well as lower intake of meat and high-fat dairy products, was associated with a lower “low-grade inflammatory score.” Shivappa et al. [60] also used a composite score called an INFLA score (including CRP, leukocytes count, and granulocyte to lymphocyte ratio), which was positively associated with DII, in 20,823 adults from the Moli-sani study. Interestingly, there was no association between food groups and CRP alone [60]. These findings emphasize the need for comprehensive and condition-specific assessment of inflammatory markers in evaluating the effects of dietary patterns.

### 4.3. Role of Weight Loss in Assessing Anti-Inflammatory Effects of Dietary Patterns

Another confounding factor in investigating the relationship between diet and inflammation is weight loss. If the interventions [22, 68, 69] include weight loss as an outcome, it may be difficult to pinpoint the actual diet as the main contributor to the reduction in inflammatory cytokines. It is well established that waist circumference is associated with CRP and that body fat reduction ameliorates low-grade inflammation [1, 70, 71]. More specifically, changes in CRP levels in response to diet have found to be dependent on weight loss [72]. Indeed, studies reporting an improvement in inflammation with diet had participants losing weight, which could have been driven by the reduction in adipose tissue mass and function [22, 55, 57, 73].

### 4.4. Fasting vs. Postprandial Effects of Diet on Inflammation

Most of the studies in the present review [22, 54, 62] reported inflammatory markers only in the fasting state. As suggested by Minihane et al. [74], assessing biomarkers in response to a dietary challenge may provide a better picture of the actual effect of the diet. We also distinguished studies measuring cytokines in the postprandial state [63] and both (postprandial and fasting) [25, 61]. For example, a study measured the effect of polyphenols from blueberry powder served in yogurt in the fasting and postprandial state. All cytokines were decreased in the postprandial state compared to the fasting state [25]. Likewise, a study examined chronic (measured at fasting) and postprandial effects of a protein mixture compared with carbohydrate intake (placebo) on inflammatory markers. Postprandial CRP levels were higher 4 hours after ingestion of the protein mix compared to maltodextrin. Meanwhile, at fasting levels after 4 weeks on the high protein diet, there were no changes in fasting plasma CRP or in SAA [61]. A recent review evaluating the effect of fruit-based drinks in postprandial studies details some risks of misinterpretation inherent to measure nutrient in the postprandial state [75].

### 4.5. Dietary Compliance

Similarly to pharmaceutical trials, a dose/effect is relevant when assessing the anti-inflammatory potential of a diet [76]. Based on the evidence presented, emphasis should be given to dietary adherence, as a lack of adherence to experimental diets may confound results and contribute to variability observed between studies. Researchers evaluating diet and inflammatory markers must address this factor by providing study participants with strategies to increase dietary adherence, while further implementing an effective procedure to measure compliance [77]. In the case of observational studies, it is crucial to have an appropriate and validated method to collect dietary intake data and a systematic way to enter and analyze the data.

### 4.6. Additional Factors

There may be a threat to the validity of results about the effect of diet in participants taking medications that affect inflammation or who have genetic variants that impact physiological and inflammatory responses to dietary components. Even though data are adjusted for confounding factors such as lipid-lowering medications, energy intake, or waist circumference, the effect of the diet may be blurred by those factors [78, 79]. Specific nutrients such as fiber or antioxidants may further exert a positive effect through their influence on the microbiota. The key role of the microbiota is not included in the scope of the present review; however, other reviews [20, 80] contain discussions on the effects of the microbiota on inflammation. The complexity of the global relationship between diet and inflammation cannot be bypassed.

## 5. Conclusions and Practical Implications

We observe vast evidence from observational studies on the correlation between a healthy dietary pattern and inflammatory markers. The high variability in the study designs and the population makes it difficult to generalize the results from the present review. Most data available on dietary patterns are observational. Ideally, though costly, conducting RCTs in healthy, overweight/obese, T2DM, and CVD individuals and measuring several biomarkers in the fasting and the postprandial state, including genotyping, would contribute to better understanding the effect diet has, as a whole, on inflammation.

Perhaps, having a proxy such as using a healthy eating score, such as the DII, could be an alternative to a more costly laboratory cytokine evaluation in clinical practice, if validated for these purposes. Comparing the patient diet with the DII score provides valuable information regarding the inflammatory potential of the diet and areas for improvement. Indeed, there is a group of researchers from South Carolina who translated their research into an educational platform, offering innovative products that can be used by health care providers and the public in order to better understand the inflammatory potential of the diet [81]. The last International Life Sciences Institute (ILSI) position paper provided a list of topics to address when building a dossier for a European Food Safety Authority health claim on control of chronic low-grade inflammation [74]. This suggests that Europe is considering adding an “inflammatory index” in labels. These trends in policy support key research findings that diet is a low-cost, preventative, and therapeutic target that must be stressed considering the role that low-grade, systemic inflammation has in highly prevalent chronic diseases.

## Figures and Tables

**Table 1:** Macronutrient distribution, dietary patterns, and inflammatory markers.

Authors, country & year	Study design/duration	Participants	Diet evaluated	Inflammatory markers/adipokines (measured in fasting plasma)	Results
Juanola-Falgarona, et al. [57] Spain, 2014	RCT, weight loss study/6 mo. GLYNDIET study	*n* = 105, men and women, aged 30 and 60 yrs. BMI range 27-35	Moderate CHO and high glycemic index vs. moderate CHO and LGI vs. a low-fat and HGI diet. 500 kcal/d subtracted of total energy for each participant	PAI-1, CRP, IL-6, MCP-1, ICAM-1, and adiponectin	(1) No differences in any of the inflammatory markers between groups. (2) A reduction in CRP values for the LGI group pre vs. postintervention, likely driven by the greater weight loss.

Jonasson et al. [55] Sweden, 2014	Clinical trial. Weight loss intervention with control group/24 mo.	*n* = 61 intervention group (T2D) and *n* = 41 control group (healthy)	LFD aiming 30% from fat vs. a LCD aiming 20% CHO	IL-1*β*, IL-1Ra, IL-6, TNFRs, and CRP at baseline and 6 mo.	(1) Both diets led to similar reductions in body weight; however, only the LCD improved insulin sensitivity. (2) After 6 mo., IL-1Ra and IL-6 were lower in the LCD.

Santiago Torres et al. [58] US, 2015	Cohort. Subset sample of the Women's Health Initiative study	*n* = 493, middle-aged women of Mexican descent	Adherence to a “created traditional Mexican diet”	hsCRP	(1) At follow-up, (15 y) hsCRP was 22% lower in women who had high compared to low or moderate MexD scores. (2) Women with high MexD scores were more likely to be normal weight at baseline.

Dias et al. [40] Sweden, 2015	Cross-sectional. Data from the Malmo Diet and Cancer study	*n* = 667, aged 63-68 yrs.	Diet quality index, adherence to the Swedish Nutrition Recommendation	IL1-beta, IL-8, TNF-*α*, and hsCRP. Mononuclear leukocytes. Inflammatory protein S100A8/9	Those who reported a higher quality diet had lower levels of TNF-*α*, hsCRP, nonclassical monocytes, and inflammatory protein S100A8/9, after adjusting for age, gender, smoking, PA, total energy, WC, and season of the diet reported.

Marques-Rocha et al. [41] Spain, 2016	RCT, weight loss intervention/8 weeks	*n* = 40, obese with MetS. Subsample of the RESMENA-S	Hypocaloric diet based on the Mediterranean diet, 30% energy restriction, increased meal frequency, and 7 d menu plan provided. Healthy Eating Index was calculated.	MDA, CRP, IL-6, PAI-1, and TNF-*α* expression of inflammation-related genes: IL-6, IL-18, TNF-*α*, and sICAM, SERPINE 1, VCAM-1 GAPDH, and miRNAs in WBC	(1) No changes in plasma CRP, IL-6, and TNF-*α*, but there was a reduction in MDA and PAI-1 after 8 weeks. (2) Lower consumption of lipids and saturated fat was associated with increased let-7b after nutritional intervention.

Song et al. [59] US, 2016	Parallel design/6 weeks. RCT feeding study	*n* = 92, healthy, aged 21-76 yrs., BMI 19.2-35.5	Eucaloric moderate fat diet (36% fat/46% CHO vs. eucaloric low-fat/high CHO (18% fat/64% CHO vs. low calories 33% reduction of the LFD)	IL-6, TNFRs, CRP, leptin, and adiponectin at baseline and 6 weeks	(1) No changes in plasma inflammatory markers after 6 weeks of MFD, LFD, and low-calorie LFD. A modest weight reduction was observed in the restricted calorie diet and adiponectin reduction was reported for the eucaloric LFD vs. MFD.

Mayr et al. [51] Australia, 2018	Multicenter, parallel design, randomized, 6-month intervention and 12-month follow-up. AUSMED Study	*n* = 29 for MedDiet and *n* = 27 for low-fat diet. Patients with CAD	MedDiet vs. low-fat diet. MedDiet = 42% fat (at least 50% was from MUFA and 25% from PUFA, <10% saturated fatty acids), 35% CHO Low-fat diet = <30% total fat, <7% saturated fat, 45–65% CHO	hsCRP and IL-6	(1) There were no changes in plasma hsCRP or IL-6 after 6 months for either diet. (2) Following the MedDiet, but not the low-fat diet, leads to significant reduction in the DII score.

Sakhaei et al. [52] Iran, 2018	Cross-sectional	*n* = 320, Yazdi female teachers, aged 25-50 yrs. Sample stratified in tertiles	Adherence to DASH diet and the Mediterranean diet based on published scoring method	hsCRP and IL-17A	Adherence to the DASH diet was significantly associated with reduced serum hsCRP only. Adherence to the Mediterranean diet might be associated with lower circulating IL-17A concentrations, but not hsCRP levels in this group of females.

Shivappa et al. [60] Italy, 2018	Cross-sectional. Data from Moli-sani study	*n* = 20.823, adults, aged over 35 yrs., 48% male without acute inflammation	DII calculated using data on only 34 nutrients and other food components derived from the FFQ.	INFLA-score includes platelet and leukocyte counts, the granulocyte to lymphocyte ratio, and CRP.	There was a positive association between DII and INFLA-score, among those aged 50 to 65 yrs., but not for those older than 65 yrs. No association between DII and CRP alone.

Abbreviations. AUSMED: Australian Mediterranean diet heart trial; DII: dietary inflammatory index; FFQ: food frequency questionnaire; HGI: high glycemic index; LCD: low carbohydrate diet; LFD: low-fat diet; LGI: low glycemic index; MedDiet: Mediterranean diet; MexD: Mexican diet; MFD: moderate-fat diet; RESMENA-S: the metabolic syndrome reduction in Navarra study; WC: waist circumference.

**Table 2:** Single nutrients and inflammatory markers.

Authors/origin/year	Study design/duration	Participants	Nutrient	Inflammatory markers measured	Results
Teunissen-Beekman et al. [61] England, 2015	(1) Postprandial study/12 H (2) Crossover, randomized parallel group design/4 weeks each. Data from the PROPRES study	(1) Postprandial study *n* = 52 (2) Crossover study *n* = 48, overweight/obese with untreated blood pressure.	(1) Postprandial responses after maltodextrin (shake) vs. sucrose vs. a specific protein mixture (pea, milk, and egg white protein). (2) Four weeks of exchanging 3 x 20g/d of CHO isoenergetically with a protein mix.	SAA, CRP, and sICAM. Fasting and postprandial (at 4 H)	(1) Postprandial CRP levels were higher 4 H after ingestion of the protein mix compared to maltodextrin, but there were no differences in sICAM and SAA. Postprandial sICAM levels were lower after pea protein vs. egg protein. (2) Significantly lower sICAM fasting levels after 4 weeks on the high-protein diet. No changes in CRP or in SAA.

Ono-Moore et al. [25] US, 2016	Placebo-controlled crossover. Postprandial	*n* = 23, normal weight, aged 27-33 yrs.	650 calories moderate high-fat breakfast (40% fat) with placebo powder or with 2 or 4 servings of the blueberry powder served in yogurt	IL-1*β*, IL-6, IL 8, and TNF-*α*. Fasting and postprandial (at 3.5 H)	There were no substantial effect of the blueberry powder on the postprandial plasma cytokines or on marker expression. All cytokines were decreased in the postprandial state compared to the fasting state; this correlates with decreased FFAs in the postprandial state.

Vitale et al. [54] Italy, 2016	Cross-sectional. Subset data from the TOSCA.IT study.	*n* = 1785, with T2D, aged 50-75 yrs.	Fiber, added sugar, and different proportions of fat and CHO.	hsCRP	Fat intake increase from 25 to 35% or more is associated with an increasing hsCRP; contrary increasing CHO 45% to 60% or more was associated with lower hsCRP. The average GI of participants' diet was low. Hs-CRP increases progressively when added sugar intake increases. Fiber intake > 15 g/1000 kcal is associated with lower hsCRP.

Cormier et al. [62] Canada, 2016	Omega-3 PUFA supplement. No control group/6 weeks. Fatty Acid Sensor Study.	*n* = 191, aged 21-39 yrs., BMI range 24-31.5.	5 g/d of fish oil supplement: 1.9-2.1 g of EPA and 1.1 of DHA	CRP, IL-1, TNF-*α*, and IL-6 gene expression in peripheral mononuclear cells	There were no reductions in plasma IL-6, CRP, or TNF-*α* postintervention. Plasma n-3 levels were negatively correlated with plasma cytokines and CRP. There were several gene-diet interactions with SNPs within inflammation-related genes and omega-3, but this varied according to individual genotypes.

Hosseini et al. [22] Iran, 2016	RCT, double-blind/30 days intervention	*n* = 48, healthy adults, aged 30-60 yrs.	500 mg pomegranate extract (PE) or placebo (PL).	MDA, IL-6, and hsCRP	The PE group lost significantly more weight than the PL. The mean serum concentration of MDA, IL-6, and hsCRP decreased significantly in the PE group. Change in body weight was correlated with these parameters.

De Lorenzo et al. [63] Italy, 2017	Crossover RCT postprandial	*n* = 22, healthy	Tocopherol-enriched Mediterranean meal (TEM), 41% CHO/16% protein/42% fat vs. Western high-fat meal (HFM), 27% CHO/18% protein/55% fat.	Genes of the inflammasome pathway and genes of the oxidative stress pathway. Fasting and postprandial (at 3 H)	A single HFM resulted in upregulation of the human inflammasome pathway genes by 15.4% and of the human oxidative pathway by 15% when compared with a TEM. Additionally, a downregulation of CCL5 was observed after the TEM vs. the HFM.

Abbreviations. MDA: malondialdehyde; PROPRES: Protein and blood pressure study; RCT: randomized, controlled trial; SAA: serum amyloid A; TOSCA.IT: thiazolidinediones or sulfonylureas and cardiovascular accident intervention trial.

## References

[1] Calle M. C., Fernandez M. L. (2012). Inflammation and type 2 diabetes. *Diabetes & Metabolism*.

[2] Wirth M. D., Burch J., Shivappa N. (2014). Association of a dietary inflammatory index with inflammatory indices and metabolic syndrome among police officers. *Journal of Occupational and Environmental Medicine*.

[3] Hotamisligil G. S. (2017). Inflammation, metaflammation and immunometabolic disorders. *Nature*.

[4] Libby P., Loscalzo J., Ridker P. M. (2018). Inflammation, immunity, and infection in atherothrombosis: JACC review topic of the week. *Journal of the American College of Cardiology*.

[5] Buttorff C., Ruder T., Bauman M. (2017). *Multiple chronic conditions in the United States*.

[6] Raghupathi W., Raghupathi V. (2018). An empirical study of chronic diseases in the United States: a visual analytics approach. *International Journal of Environmental Research and Public Health*.

[7] Petersen A. M. W., Pedersen B. K. (2005). The anti-inflammatory effect of exercise. *Journal of Applied Physiology*.

[8] Ridker P. M. (2007). C-reactive protein and the prediction of cardiovascular events among those at intermediate risk: moving an inflammatory hypothesis toward consensus. *Journal of the American College of Cardiology*.

[9] Demerath E., Towne B., Blangero J., Siervogel R. M. (2001). The relationship of soluble ICAM-1, VCAM-1, P-selectin and E-selectin to cardiovascular disease risk factors in healthy men and women. *Annals of Human Biology*.

[10] Mozos I., Malainer C., Horbańczuk J. (2017). Inflammatory markers for arterial stiffness in cardiovascular diseases. *Frontiers in Immunology*.

[11] Andersen C. J., Murphy K. E., Fernandez M. L. (2016). Impact of obesity and metabolic syndrome on immunity. *Advances in Nutrition*.

[12] Rivera A., Siracusa M. C., Yap G. S., Gause W. C. (2016). Innate cell communication kick-starts pathogen-specific immunity. *Nature Immunology*.

[13] den Haan J. M. M., Arens R., van Zelm M. C. (2014). The activation of the adaptive immune system: cross-talk between antigen-presenting cells, T cells and B cells. *Immunology Letters*.

[14] Schett G., Neurath M. F. (2018). Resolution of chronic inflammatory disease: universal and tissue-specific concepts. *Nature Communications*.

[15] Sun K., Kusminski C. M., Scherer P. E. (2011). Adipose tissue remodeling and obesity. *The Journal of Clinical Investigation*.

[16] Guilherme A., Virbasius J. V., Puri V., Czech M. P. (2008). Adipocyte dysfunctions linking obesity to insulin resistance and type 2 diabetes. *Nature Reviews Molecular Cell Biology*.

[17] Andersen C. J., Fernandez M. L. (2013). Dietary strategies to reduce metabolic syndrome. *Reviews in Endocrine & Metabolic Disorders*.

[18] Kirwan A. M., Lenighan Y. M., O'Reilly M. E., McGillicuddy F. C., Roche H. M. (2017). Nutritional modulation of metabolic inflammation. *Biochemical Society Transactions*.

[19] Egger G., Dixon J. (2010). Inflammatory effects of nutritional stimuli: further support for the need for a big picture approach to tackling obesity and chronic disease. *Obesity Reviews*.

[20] Kuo S. M. (2013). The interplay between fiber and the intestinal microbiome in the inflammatory response. *Advances in Nutrition*.

[21] Fritsche K. L. (2015). The science of fatty acids and inflammation. *Advances in Nutrition*.

[22] Hosseini B., Saedisomeolia A., Wood L. G., Yaseri M., Tavasoli S. (2016). Effects of pomegranate extract supplementation on inflammation in overweight and obese individuals: a randomized controlled clinical trial. *Complementary Therapies in Clinical Practice*.

[23] Dietary, U.S.D.o.H.a.H.S.a.U.S.D.o.A.-. and G.f.A.R.f. https://www.cnpp.usda.gov/2015-2020-dietary-guidelines-americans.

[24] Andersen C. (2015). Bioactive egg components and inflammation. *Nutrients*.

[25] Ono-Moore K. D., Snodgrass R. G., Huang S. (2016). Postprandial inflammatory responses and free fatty acids in plasma of adults who consumed a moderately high-fat breakfast with and without blueberry powder in a randomized placebo-controlled trial. *The Journal of Nutrition*.

[26] Ahluwalia N., Andreeva V. A., Kesse-Guyot E., Hercberg S. (2013). Dietary patterns, inflammation and the metabolic syndrome. *Diabetes & Metabolism*.

[27] Calle M. C., Vega-López S., Segura-Pérez S., Pérez-Escamilla R., Fernandez M. L. (2013). Dietary pattern low in fruits explains variations in inflammation and in biomarkers of cardiovascular disease in Latinos diagnosed with type-2 diabetes. *British Journal of Medicine and Medical Research*.

[28] Jacobs D. R., Steffen L. M. (2003). Nutrients, foods, and dietary patterns as exposures in research: a framework for food synergy. *The American Journal of Clinical Nutrition*.

[29] Reedy J., Krebs-Smith S. M., Hammond R. A., Hennessy E. (2017). Advancing the science of dietary patterns research to leverage a complex systems approach. *Journal of the Academy of Nutrition and Dietetics*.

[30] Fung T. T., Rimm E. B., Spiegelman D. (2001). Association between dietary patterns and plasma biomarkers of obesity and cardiovascular disease risk. *The American Journal of Clinical Nutrition*.

[31] Lopez-Garcia E., Schulze M. B., Fung T. T. (2004). Major dietary patterns are related to plasma concentrations of markers of inflammation and endothelial dysfunction. *The American Journal of Clinical Nutrition*.

[32] Poggio R., Elorriaga N., Gutierrez L., Irazola V., Rubinstein A., Danaei G. (2017). Associations between dietary patterns and serum lipids, apo and C-reactive protein in an adult population: evidence from a multi-city cohort in South America. *The British Journal of Nutrition*.

[33] Vitale M., Masulli M., Calabrese I. (2018). Impact of a Mediterranean dietary pattern and its components on cardiovascular risk factors, glucose control, and body weight in people with type 2 diabetes: a real-life study. *Nutrients*.

[34] Stewart R. A. H. (2018). Primary prevention of cardiovascular disease with a Mediterranean diet supplemented with extra-virgin olive oil or nuts. *The New England Journal of Medicine*.

[35] Waldeyer C., Brunner F. J., Braetz J. (2018). Adherence to Mediterranean diet, high-sensitive C-reactive protein, and severity of coronary artery disease: contemporary data from the INTERCATH cohort. *Atherosclerosis*.

[36] Tabung F. K., Smith-Warner S. A., Chavarro J. E. (2016). Development and validation of an empirical dietary inflammatory index. *The Journal of Nutrition*.

[37] Shivappa N., Steck S. E., Hurley T. G., Hussey J. R., Hébert J. R. (2014). Designing and developing a literature-derived, population-based dietary inflammatory index. *Public Health Nutrition*.

[38] Schwingshackl L., Hoffmann G. (2015). Diet quality as assessed by the healthy eating index, the alternate healthy eating index, the dietary approaches to stop hypertension score, and health outcomes: a systematic review and meta-analysis of cohort studies. *Journal of the Academy of Nutrition and Dietetics*.

[39] Tabung F. K., Steck S. E., Zhang J. (2015). Construct validation of the dietary inflammatory index among postmenopausal women. *Annals of Epidemiology*.

[40] Dias J. A., Wirfält E., Drake I. (2015). A high quality diet is associated with reduced systemic inflammation in middle-aged individuals. *Atherosclerosis*.

[41] Marques-Rocha J. L., Milagro F. I., Mansego M. L., Zulet M. A., Bressan J., Martínez J. A. (2016). Expression of inflammation-related miRNAs in white blood cells from subjects with metabolic syndrome after 8 wk of following a Mediterranean diet-based weight loss program. *Nutrition*.

[42] Shivappa N., Hébert J. R., Rietzschel E. R. (2015). Associations between dietary inflammatory index and inflammatory markers in the Asklepios study. *The British Journal of Nutrition*.

[43] Tabung F. K., Giovannucci E. L., Giulianini F. (2018). An empirical dietary inflammatory pattern score is associated with circulating inflammatory biomarkers in a multi-ethnic population of postmenopausal women in the United States. *The Journal of Nutrition*.

[44] Soltani S., Moslehi N., Hosseini-Esfahani F., Vafa M. (2018). The association between empirical dietary inflammatory pattern and metabolic phenotypes in overweight/obese adults. *International Journal of Endocrinology and Metabolism*.

[45] Wirth M. D., Burch J., Shivappa N. (2014). Dietary inflammatory index scores differ by shift work status: NHANES 2005 to 2010. *Journal of Occupational and Environmental Medicine*.

[46] Bodén S., Wennberg M., van Guelpen B. (2017). Dietary inflammatory index and risk of first myocardial infarction; a prospective population-based study. *Nutrition Journal*.

[47] Garcia-Arellano A., Martínez-González M. A., Ramallal R. (2018). Dietary inflammatory index and all-cause mortality in large cohorts: the SUN and PREDIMED studies. *Clinical Nutrition*.

[48] Jorgensen D., White G. E., Sekikawa A., Gianaros P. (2018). Higher dietary inflammation is associated with increased odds of depression independent of Framingham risk score in the national health and nutrition examination survey. *Nutrition Research*.

[49] Phillips C. M., Shivappa N., Hébert J. R., Perry I. J. (2018). Dietary inflammatory index and biomarkers of lipoprotein metabolism, inflammation and glucose homeostasis in adults. *Nutrients*.

[50] Farhangi M. A., Najafi M. (2018). Dietary inflammatory index: a potent association with cardiovascular risk factors among patients candidate for coronary artery bypass grafting (CABG) surgery. *Nutrition Journal*.

[51] Mayr H. L., Thomas C. J., Tierney A. C. (2018). Randomization to 6-month Mediterranean diet compared with a low-fat diet leads to improvement in dietary inflammatory index scores in patients with coronary heart disease: the AUSMED heart trial. *Nutrition Research*.

[52] Sakhaei R., Shahvazi S., Mozaffari-Khosravi H. (2018). The dietary approaches to stop hypertension (DASH)-style diet and an alternative Mediterranean diet are differently associated with serum inflammatory markers in female adults. *Food and Nutrition Bulletin*.

[53] Bonaccio M., Cerletti C., Iacoviello L., de Gaetano G. (2015). Mediterranean diet and low-grade subclinical inflammation: the Moli-sani study. *Endocrine, Metabolic & Immune Disorders Drug Targets*.

[54] Vitale M., Masulli M., Rivellese A. A. (2016). Influence of dietary fat and carbohydrates proportions on plasma lipids, glucose control and low-grade inflammation in patients with type 2 diabetes-the TOSCA.IT study. *European Journal of Nutrition*.

[55] Jonasson L., Guldbrand H., Lundberg A. K., Nystrom F. H. (2014). Advice to follow a low-carbohydrate diet has a favourable impact on low-grade inflammation in type 2 diabetes compared with advice to follow a low-fat diet. *Annals of Medicine*.

[56] Raygan F., Bahmani F., Kouchaki E. (2016). Comparative effects of carbohydrate versus fat restriction on metabolic profiles, biomarkers of inflammation and oxidative stress in overweight patients with type 2 diabetic and coronary heart disease: a randomized clinical trial. *ARYA Atherosclerosis*.

[57] Juanola-Falgarona M., Salas-Salvadó J., Ibarrola-Jurado N. (2014). Effect of the glycemic index of the diet on weight loss, modulation of satiety, inflammation, and other metabolic risk factors: a randomized controlled trial. *The American Journal of Clinical Nutrition*.

[58] Santiago-Torres M., Tinker L. F., Allison M. A. (2015). Development and use of a traditional Mexican diet score in relation to systemic inflammation and insulin resistance among women of Mexican descent. *The Journal of Nutrition*.

[59] Song X., Kestin M., Schwarz Y. (2016). A low-fat high-carbohydrate diet reduces plasma total adiponectin concentrations compared to a moderate-fat diet with no impact on biomarkers of systemic inflammation in a randomized controlled feeding study. *European Journal of Nutrition*.

[60] Shivappa N., Bonaccio M., Hebert J. R. (2018). Association of proinflammatory diet with low-grade inflammation: results from the Moli-sani study. *Nutrition*.

[61] Teunissen-Beekman K. F. M., Dopheide J., Geleijnse J. M. (2015). Dietary proteins improve endothelial function under fasting conditions but not in the postprandial state, with no effects on markers of low-grade inflammation. *The British Journal of Nutrition*.

[62] Cormier H., Rudkowska I., Lemieux S., Couture P., Vohl M. C. (2016). Expression and sequence variants of inflammatory genes; effects on plasma inflammation biomarkers following a 6-week supplementation with fish oil. *International Journal of Molecular Sciences*.

[63] De Lorenzo A., Bernardini S., Gualtieri P. (2017). Mediterranean meal versus Western meal effects on postprandial ox-LDL, oxidative and inflammatory gene expression in healthy subjects: a randomized controlled trial for nutrigenomic approach in cardiometabolic risk. *Acta Diabetologica*.

[64] Monfort-Pires M., Ferreira S. R. G. (2017). Inflammatory and metabolic responses to dietary intervention differ among individuals at distinct cardiometabolic risk levels. *Nutrition*.

[65] Ratliff J. C., Mutungi G., Puglisi M. J., Volek J. S., Fernandez M. (2008). Eggs modulate the inflammatory response to carbohydrate restricted diets in overweight men. *Nutrition & Metabolism*.

[66] Calder P. C., Ahluwalia N., Albers R. (2013). A consideration of biomarkers to be used for evaluation of inflammation in human nutritional studies. *The British Journal of Nutrition*.

[67] van Bussel B. C., Henry R. M. A., Ferreira I. (2015). A healthy diet is associated with less endothelial dysfunction and less low-grade inflammation over a 7-year period in adults at risk of cardiovascular disease. *The Journal of Nutrition*.

[68] Dahlberg C. J., Ou J. J., Babish J. G. (2017). A 13-week low glycemic load diet and lifestyle modification program combining low glycemic load protein shakes and targeted nutraceuticals improved weight loss and cardio-metabolic risk factors. *Canadian Journal of Physiology and Pharmacology*.

[69] Merra G., Gratteri S., de Lorenzo A. (2017). Effects of very-low-calorie diet on body composition, metabolic state, and genes expression: a randomized double-blind placebo-controlled trial. *European Review for Medical and Pharmacological Sciences*.

[70] Ackermann D., Jones J., Barona J. (2011). Waist circumference is positively correlated with markers of inflammation and negatively with adiponectin in women with metabolic syndrome. *Nutrition Research*.

[71] Choi J., Joseph L., Pilote L. (2013). Obesity and C-reactive protein in various populations: a systematic review and meta-analysis. *Obesity Reviews*.

[72] Steckhan N., Hohmann C. D., Kessler C., Dobos G., Michalsen A., Cramer H. (2016). Effects of different dietary approaches on inflammatory markers in patients with metabolic syndrome: a systematic review and meta-analysis. *Nutrition*.

[73] Magkos F., Fraterrigo G., Yoshino J. (2016). Effects of moderate and subsequent progressive weight loss on metabolic function and adipose tissue biology in humans with obesity. *Cell Metabolism*.

[74] Minihane A. M., Vinoy S., Russell W. R. (2015). Low-grade inflammation, diet composition and health: current research evidence and Its translation. *The British Journal of Nutrition*.

[75] Peluso I., Palmery M. (2014). Risks of misinterpretation in the evaluation of the effect of fruit-based drinks in postprandial studies. *Gastroenterology Research and Practice*.

[76] Szulińska M., Łoniewski I., van Hemert S., Sobieska M., Bogdański P. (2018). Dose-dependent effects of multispecies probiotic supplementation on the lipopolysaccharide (LPS) level and cardiometabolic profile in obese postmenopausal women: a 12-week randomized clinical trial. *Nutrients*.

[77] Gibson A. A., Sainsbury A. (2017). Strategies to improve adherence to dietary weight loss interventions in research and real-world settings. *Behavioral Sciences*.

[78] Christian A. H., Mochari H., Mosca L. J. (2009). Waist circumference, body mass index, and their association with cardiometabolic and global risk. *Journal of the Cardiometabolic Syndrome*.

[79] Winkvist A., Bärebring L., Gjertsson I., Ellegård L., Lindqvist H. M. (2018). A randomized controlled cross-over trial investigating the effect of anti-inflammatory diet on disease activity and quality of life in rheumatoid arthritis: the anti-inflammatory diet in rheumatoid arthritis (ADIRA) study protocol. *Nutrition Journal*.

[80] Andoh A. (2016). Physiological role of gut microbiota for maintaining human health. *Digestion*.

[81] Connecting Health Innovations. 2018 [cited 2018; DII]. http://chi-llc.net.

