# Corrigendum to “Assessment of Dietary Patterns Represents a Potential, Yet Variable, Measure of Inflammatory Status: A Review and Update”

**DOI:** 10.1155/2019/5454602

**Published:** 2019-09-03

**Authors:** Mariana C. Calle, Catherine J. Andersen

**Affiliations:** ^1^Health Sciences Department, Worcester State University, Worcester, MA 01602, USA; ^2^Department of Biology, Fairfield University, Fairfield, CT 06824, USA

In the article titled “Assessment of Dietary Patterns Represents a Potential, Yet Variable, Measure of Inflammatory Status: A Review and Update” [1], the authors did not use the most updated acronym when referring to the Empirical Dietary Inflammatory Index (EDII) [2], as it is now referred to as the Empirical Dietary Inflammatory Pattern (EDIP) [3], which is distinct from the energy-adjusted DII® (E-DII).

In Section 2.3, Diet Inflammatory Index (DII), the authors mentioned that the Dietary Inflammatory Index (DII) uses a principal component analysis to categorize an individual's diet as anti- or proinflammatory, based on the capacity of diets to modulate systemic inflammatory biomarkers. However, it was not derived by using principal component analysis. This should be corrected as follows:

“The population-based DII® represents a refined scoring algorithm based on extensive review of the literature and construction of a global reference database to assess the inflammatory potential of the diet [4].”
